# Medical Inspection

**Published:** 1916-03

**Authors:** 


### Medical Inspection.

Dr. J. H. Smart had a sensible article in the *Dallas News* recently urging medical inspection of the students attending public schools. Whether it is wanted or not the compulsory education law will lead to this. Where children are compelled to attend school they will be under the necessity of securing medical certificates to excuse their absence, and from that there will be but a step to certificates permitting them to attend school and mingle with other children.

Medical inspection along other lines is needed as badly as for school children. We are compelled to recognize the fact that public attention is directed as never before to preventive measures. There should be boards of specialists whose duty it should be to inspect stagnant pools of water that are the hatching places from which start many of our worst diseases; to examine barns and other places where filth quickly accumulates and flies spring forth by the thousands; to see that the foods are properly prepared and handled; in short, to see that the public health is properly safeguarded. The State is doing something along these lines already, but its work cannot prove effective without local co-operation.

There is not a city in Texas where insanitary conditions do not exist that are a disgrace to our civilization. These places are found everywhere, and they will continue to exist until physicians as a body become active in remedying the evil. So long as the medical fraternity shows indifference the general public feels that there is no need for action.

